# Both selective and neutral processes drive GC content evolution in the human genome

**DOI:** 10.1186/1471-2148-8-99

**Published:** 2008-03-27

**Authors:** Uberto Pozzoli, Giorgia Menozzi, Matteo Fumagalli, Matteo Cereda, Giacomo P Comi, Rachele Cagliani, Nereo Bresolin, Manuela Sironi

**Affiliations:** 1 Scientific Institute IRCCS E. Medea, Bioinformatic Lab, Via don L. Monza 20, 23842 Bosisio Parini (LC), Italy; 2 Dino Ferrari Centre, Department of Neurological Sciences, University of Milan, IRCCS Ospedale Maggiore Policlinico, Mangiagalli and Regina Elena Foundation, Via F. Sforza 35, 20100 Milan, Italy

## Abstract

**Background:**

Mammalian genomes consist of regions differing in GC content, referred to as isochores or GC-content domains. The scientific debate is still open as to whether such compositional heterogeneity is a selected or neutral trait.

**Results:**

Here we analyze SNP allele frequencies, retrotransposon insertion polymorphisms (RIPs), as well as fixed substitutions accumulated in the human lineage since its divergence from chimpanzee to indicate that biased gene conversion (BGC) has been playing a role in within-genome GC content variation. Yet, a distinct contribution to GC content evolution is accounted for by a selective process. Accordingly, we searched for independent evidences that GC content distribution does not conform to neutral expectations. Indeed, after correcting for possible biases, we show that intron GC content and size display isochore-specific correlations.

**Conclusion:**

We consider that the more parsimonious explanation for our results is that GC content is subjected to the action of both weak selection and BGC in the human genome with features such as nucleosome positioning or chromatin conformation possibly representing the final target of selective processes. This view might reconcile previous contrasting findings and add some theoretical background to recent evidences suggesting that GC content domains display different behaviors with respect to highly regulated biological processes such as developmentally-stage related gene expression and programmed replication timing during neural stem cell differentiation.

## Background

Mammalian genomes are non homogeneous with respect to base composition; striking variations in GC content occur over scales of hundred kilobases to megabases. The so called isochoric structure of the human genome was initially described by Bernardi and coworkers [1] and isochores were conceived as long genomic regions fairly homogeneous in their GC composition. Full sequencing of the human genome [2] indicated that the isochore model might need slight revision in that long regions are less compositionally homogeneous than previously thought and transitions at composition domains less sharp, so that the term "GC-content domain" was proposed instead of "isochore". Whatever designation we decide to adopt, the fact remains that isochores/GC content domains represent a large-scale genomic feature lacking a satisfactory interpretation. Indeed, the scientific debate is still open as to whether such a compositional heterogeneity is a selected or neutral trait and different hypothesis have been proposed [3,4]. The biased gene conversion (BGC) model [5,6] envisages a situation whereby recombination drives GC content in mammalian genomes through the preferential fixation of GC alleles following parental chromosome hetroduplex formation at meiosis. The effect is due to the bias toward GC nucleotides over AT during DNA repair at mismatched bases [7]. The model therefore conceives of GC content variation as a by-product of recombination and, although supported by extensive evidence [8-13], its ability to explain isochore formation and maintenance has recently been criticized on different grounds. Spencer et al. [14] have indicated that recombination rates are too fast-evolving to have permanent effects on base composition; the authors therefore suggested that the cause-consequence relationships might be the other way round with GC rich regions promoting the occurrence of recombination hotspots. Also, several studies have suggested that GC content variation results from a selective process [15-20]. In particular, a role for GC content in chromatin organization and, therefore, gene regulation has been proposed [16,18,19]. Indeed, GC content has been shown to covary with genomic properties such as regulated replication or expression timing [21,22], DNA bendability [15] and ability to B-Z transition [23], while the existence of a relationship between gene expression level (or breadth) and GC content is still controversial [9,24,25]. Nonetheless, a positive effect of increased coding sequence GC content on transcriptional efficiency has recently been experimentally demonstrated [25].

Up to now, with the exception of the above mentioned studies on gene expression, evidences of selection acting on GC content *per se *have been scant (see [3] for review). This might partially be due to difficulty in discriminating between BGC and weak selection.

Here we analyze SNP allele frequencies, retrotransposon insertion polymorphisms (RIPs), as well as fixed substitutions accumulated in the human lineage since its divergence from chimpanzee to indicate that both biased gene conversion (BGC) and selection have been playing a role in GC content variation.

## Methods

### Data retrieval

Gene and intergenic sequences as well as intron/exon boundaries were obtained from the UCSC genome annotation database [26], assembly hg17. Gene selection was performed as previously described [27]. Isochore boundary coordinates were derived from a previous work [20]. Fine-scale recombination rates and recombination hotspot locations were obtained from the UCSC database; they are based on HapMap Phase I data [28]. Pseudogene sequences and genomic locations derive from Pseudogene.org [29,30]; only duplicated pseudogenes were selected and genes that generated more than one pseudogene were discarded (this procedure limits the number of observations but avoids multiple ties in statistical analysis). Also we retained only gene-pseudogenes pairs located in the same isochore type (for example, both gene and pseudogene located in isochores H1). The final data set consisted of 364 gene-pseudogene pairs. Duplicated pseudogenes often represent gene fragments; we therefore aligned gene-pseudogene couples using ClustalW [31] and corresponding intron-pseudointron pairs were retained only if they were both longer than 25 bp. Expression data were obtained as previously described [27] and derive from microarray data on 72 healthy human tissues. Mean expression level was calculated as the mean averaged over all tissues (counting as zero all tissues in which there is no detectable expression). Peak expression was calculated as the maximum expression level across all tissues and expression breadth was the number of distinct tissues expressing the gene.

### Polymorphism data

Biallelic SNP locations and allele frequencies were downloaded from the HapMap web site [28] (non-redundant dataset, release 21a). Since previous authors [32] have indicated African populations as having genetic variation patterns most compatible with a constant population size, SNP allele frequencies were obtained for Yoruba (YRI), and derive from the genotyping of 60 individuals. The ancestral allele was inferred by alignment with the chimpanzee sequence (UCSC genome browser, assembly panTro1); SNPs were discarded when orthologous chimpanzee regions were unavailable or did not match either human allele. A total of about 2.2 million GC->AT and 1.7 million AT->GC SNPs were retained. We next purged SNPs at CpG sites, as well as those with no associated allele frequencies: the final dataset comprised more than 2 million SNPs.

For the analysis of substitution rates and stationary GC content (GC*), SNPs deriving from the Seattle SNP database [33], which derive from resequencing experiments, were used; for 206 human genes in the Seattle SNP dataset both chimpanzee and macaque orthologous loci could be retrieved.

Data on polymorphic repeat insertions were obtained through the UCSC genome database (RIPs track) and derive from the dbRIP database [34]; RIPs which have been associated with a human genetic disease were discarded. Also, polymorphic insertions were not included in the study if less than 10 instances were described for the same retrotransposon subfamily. Fixed transposon instances were identified and categorized using the UCSC annotation tables that rely on RepeatMasker. Since fixed and polymorphic repeat instances derive from different sources, we verified that no systematic bias occurs in the detection of either insertion events by calculating correlation between polymorphic and fixed chromosomal frequencies; significant correlations were retrieved for Alus, SVAs and L1s (Spearman rho = 0.854, 0.439 and 0.923, respectively; all *p *values < 0.05). Reference sequences for different retrotransposon subtypes were derived from Repbase Update [35,36].

### Analysis of allele frequency spectra

Introns/intergenic spacers were divided in 1 kb windows (1 kbseqs) starting from the most 5' nucleotide position (with respect to the chromosome orientation) and extending through the intron/intergenic region in 1000 bp non-overlapping steps (residual nucleotides in 3' were discarded). The following features were then calculated (or retrieved) for all 1 kbseqs: (1) fine scale recombination rate, (2) GC content, (3) allele frequencies of comprised SNPs, (4) expression parameters (peak, mean level and breadth) of the corresponding genes. In order to analyze allele frequency spectra after controlling for recombination rate, we applied the following procedure: starting from all 1 kbseqs, we identified couples of 1 kbseqs that differed less than 10% in recombination rate but displayed extremely different GC contents; in particular, we asked one partner of the recombination-coupled 1 kbseqs to be located below the 30^th ^percentile in the distribution of 1 kbseqs GC content and the other one above the 70^th ^percentile. This approach yielded two groups of sequences having extremely similar recombination rates (the equality of medians was checked using the Wilcoxon Rank Sum Test) but very different GC contents. A similar procedure was applied to analyze allele frequency spectra after controlling for recombination rate; in particular, 1 kbseq couples were created having similar GC content (a difference lower than 5% was required) but extremely different recombination rates. Again, two groups of sequences were obtained and used for comparisons.

The same approach described above can be extended to control for two variables: for example, in order to compare allele frequency spectra between highly and lowly expressed sequences, 1 kbseqs couples were identified that displayed both similar GC content and recombination rate (less than 5% and 10% difference, respectively) but extremely different expression levels. To allow comparisons between introns and intergenic spacers, percentile values were calculated over the complete set of 1 kbseqs, irrespective of their location.

In order to quantify the displacement of GC vs AT derived allele frequency distributions observed in Quantile-Quantile plots, differences between corresponding percentiles in the two distributions were summed. These measures were used to compare different groups of sequences selected on the basis of relevant variables (for example high and low GC content or recombination rate). We used bootstrapping procedures to assess the statistical significance of differences in allele frequencies shifts. In particular, 1000 permutations were performed and *p *values were calculated after normality assessment through the Shapiro-Wilk Test.

### Multispecies alignments, substitution rates and stationary GC content

Orthologous human-chimpanzee-macaque regions were retrieved using the liftOver utility from UCSC (assemblies: panTro1 and rheMac2) with a cutoff of at least 70% remapping bases. Three-way species alignments were performed using MAVID [37].

In order to calculate substitution rates and GC* after controlling for ancestral GC content or recombination rate, a procedure similar to the one described above for SNP allele frequencies was applied, with the only difference that the inferred ancestral GC content was used instead of human GC content. In particular, 1 kbseqs couples were created (on the basis of either recombination rate or ancestral GC content) and their position subsequently mapped onto the 3-way species (human/chimpanzee/macaque) alignments; at this stage windows containing less than 600 perfectly aligning bases (i.e. the same nucleotide in the 3 species) were discarded and, for the remaining ones, the ancestral sequence was reconstructed by parsimony (only positions where the macaque was identical to either human or chimpanzee were considered).

The number of 1 kbseqs couples and the corresponding number of sites (in MB) that were analyzed for each comparison are reported in table notes. The number of sites does not exactly correspond to the number of sequences multiplied by 1000 because the presence of gaps in the human sequence (as compared to the two primates) can result in alignments longer than 1000 bp.

Substitution rates and stationary GC content were calculated using a previously developed neighbor-dependent substitution model [38,39]. For each comparison, the two 1 kbseqs groups were then divided in 20 paired sub-samples of equal size; GC* and substitution rates were calculated for each sub-sample; average values are reported in the tables, together with *p *values obtained from two tailed Wilcoxon Rank Sum Tests for paired samples.

For the analysis of intron GC content in relation to size, we discarded first introns (due to their increased sequence constraints) and introns shorter than 750 bp (in order to spare constrained sequences at splice sites).

For the analysis, of recombination rates in long and short introns, for each gene, two introns were selected so that one was longer than 80^th ^and the other shorter than 20^th ^size percentile of introns length distribution. If no introns satisfied the criteria, the gene was not analyzed. Recombination rates were calculated for 500 bp centered around the median position of each intron. Differences in recombination rates were evaluated using the Wilcoxon Rank Sum Test.

For the analysis of fixed variations in recombination hotspots, we selected 897 hotspot on the basis of their size (smaller than 5 kb) and recombination rate (above the 80^th ^percentile of the distribution of all hotspots); in 790 cases both chimpanzee and macaque orthologous regions could be retrieved.

### Statistical analysis

All statistical analysis were performed using R [40]. For loess fittings [41] a smoothing span of 0.5 was used.

## Results and Discussion

### Analysis of SNP allele frequencies

The analysis of SNP allele frequencies is a convenient strategy to study GC content evolution for two main reasons. First, when SNP allele frequencies are analyzed, no requirement for base composition stationarity is needed; this is relevant since base composition has been shown not to be at equilibrium in mammals [42,43]. Second, given the fast evolution of recombination rates and hotspots [44,45], allele frequencies of SNPs, which represent relatively recent variations, should carry the most evident signature of recombination-associated fixation biases.

Starting from our gene set, we therefore used the chimpanzee sequence to infer the ancestral allele so that variations could be classified as either GC->AT or AT->GC (SNPs at CpG sites were excluded). As previously noted [43] treating SNPs as independent data, despite the extensive presence of linkage disequilibrium in the human genome [42], introduces no bias since linkage is expected to be independent from the GC/AT status of individual SNPs.

**Figure 1:**
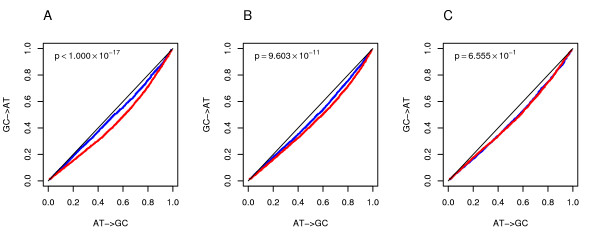
**Comparison of allele frequency spectra**. (**A**) Quantile-quantile plots of GC->AT and AT-> GC derived allele frequencies for highly (red) and low (blue) recombining intronic regions after fixing GC content. (**B**) The same as (**A**), but in this case we fixed recombination rates and compared high (red) vs low (blue) GC regions. (**C**) The same as (**A**), but in this case we fixed both GC content and recombination rates in order to compare regions from highly (red) vs low (blue) expressed genes.

BGC and selection are both expected to result in AT->GC mutations segregating at higher frequencies compared to GC->AT. Yet, this effect is expected to be stronger in highly recombining regions if BGC is involved. Conversely, selection on GC content should be acting differentially depending on the background GC content of SNP flanking regions; in particular, AT->GC variations are expected to segregate at higher frequency in GC rich regions, irrespective of recombination rate. In order to disentangle a possible selective effect from BGC, we analyzed SNP allele frequencies in noncoding genomic sequences after correcting for either GC content or recombination rate. As further detailed in methods, genomic regions were divided in 1 kb sub-sequences. These latter were arranged in couples having very similar recombination rate and extremely different GC content for the comparison of allele frequencies between GC-rich and -poor sequences. Similarly, for the comparison between high- and low-recombining sequences, sequences were arranged in couples showing very similar GC content but extremely different recombination rates. The results of SNP frequency spectra analysis are reported in figure 1 as quantile-quantile plots; in agreement with previous findings [14] and consistent with the action of BGC, GC derived alleles display higher frequencies than AT alleles but the effect is significantly (*p *< 10^-17^) stronger in highly recombining regions for both introns (Figure 1A) and intergenic spacers (see Additional file 1); yet, when allele frequencies were compared after fixing recombination rate, a residual effect of GC content was observed: derived GC alleles segregate at significantly higher frequencies in regions showing a high GC content (*p *= 2.07 × 10^-10^) compared to AT-rich regions (Figure 1B and Additional file 1 for intergenic spacers).

These data suggest that GC content or other related features affect SNP allele segregation independently of recombination rates, although we cannot formally rule out the possibility that extinct recombination hotspots have played a role in the allele frequency spectra we observe. Indeed, as reported above, recombination hotspots are fast evolving [44,45] and, therefore, the observed increased segregation of GC alleles in GC-rich regions might have been caused by the presence of an hotspot which is now inactive. Yet, if this latter were the case, given the relatively small effect that recombination has played in recent primate history on GC variation (see below), and given that most SNPs are specific to humans (and, therefore, relatively young), a direct role for GC content in promoting recombination events must be postulated to explain our results.

Since GC content has been shown to increase transcriptional activity [46] and some authors detected a positive correlation between gene expression parameters and GC content [16,18,19,46], we wished to determine whether expression level, rather than GC content *per se*, was responsible for increased segregation of GC alleles. Yet, after controlling for both GC content and recombination rates (as described in methods, we used a similar approach to the one described above) we detected no significant difference in SNP allele frequencies between high- and low-level expressed genes (Figure 1c and Additional file 1). These data are not consistent with selection acting on highly or broadly expressed human genes to increase (or maintain) their GC content, although we cannot exclude that such a selection has acted during vertebrate evolution and subsequently relaxed in humans (further data on gene expression level and GC content evolution are reported below).

### Analysis of retrotransposon insertion polymorphisms

**Figure 2:**
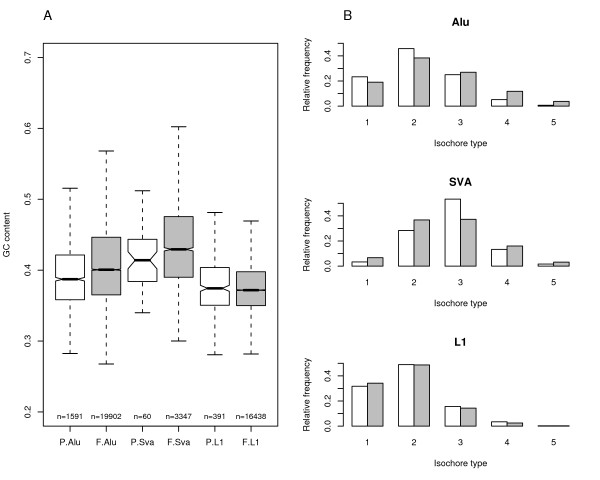
**Analysis of fixed versus polymorphic retrotransposon insertions**. (A) Analysis of average GC content flanking polymorphic (P, white) and fixed (F, gray) retrotransposons. GC content was calculated in 5 kb flanking the repeat. The number of repeat instances is also indicated. GC content is significantly higher for regions flanking fixed compared to polymorphic Alus ; the same holds for SVAs. (**B**) Analysis of polymorphic (white) and fixed (gray) retrotransposon relative frequency in different isochores (L1 to H3, ordered from 1 to 5, as described in [20]). Fixed Alus are significantly enriched in heavy isochores compared to polymorphic instances.

It has been suggested [3] that, if selection is acting on base composition, it should also affect the fixation probabilities of transposable elements; indeed, fixed Alu and LINE-1 (L1) elements (average GC content of reference sequences = 0.53 and 0.41, respectively) are differentially represented in the human genome depending on GC content [2], despite both having a preference for AT-rich integration sites [47,48]. Yet, a better estimation of fixation versus integration probabilities might be obtained by the comparison of polymorphic and fixed transposable elements. We retrieved all available instances of retrotransopson insertion polymorphisms. For both Alu and L1 repeats, we restricted the analysis of fixed repeats to the same subfamilies showing at least 10 instances of polymorphic insertions. Such subfamilies represent relatively young insertion events, yet, given the previously reported preference of older Alus for GC rich regions [2,49] we further purged all fixed Alu elements showing a divergence higher than 5%. As shown in figure 2A, both SVA (GC content of reference sequence = 0.63) and Alu fixed elements are located in regions with significantly higher GC content compared to their polymorphic counterparts (Wilcoxon Rank Sum Test, two-tailed, p = 0.028 and p < 10^-21^, respectively); conversely, fixed and polymorphic L1 flanking regions do not show different average GC contents, being both relatively GC poor (as L1 sequences are).

We next wished to verify whether polymorphic and fixed repetitive elements were differently distributed depending on isochore type. Isochores were classified according to a recent [20] description and are referred to as L1, L2, H1, H2, and H3, in order of increasing GC levels. The results of transposable element distribution are reported in figure 2B and indicate that fixed Alus are significantly enriched (Chi Square Test, p < 10^-5^) within heavy isochores compared to polymorphic instances, while no different isochore distribution of fixed vs polymorphic repeats was evident for SVAs (possibly because of the small number of polymorphic insertions, *n *= 60) or L1s. For further confirmation we performed this same analysis using IsoFinder isochores [50] and the same results were obtained (see Additional file 2). These data confirm the preferential integration of Alus and L1s in AT-rich regions (polymorphic L1 and Alu distributions are relatively similar, Figure 2B), but indicate that additional forces, which relate to GC content, drive their fixation.

We believe that our results differ from previous reports showing no different GC content surrounding polymorphic and fixed Alus [51,52] because of the larger sample of polymorphic elements we analyzed.

### Analysis of substitution rates and stationary GC content

We next wished to infer nucleotide changes fixed in the human lineage after divergence from chimpanzee by using macaque as an outgroup. In analogy to the procedure we applied for SNP frequency spectra, we analyzed substitution rates and stationary GC content (GC*, i.e. the GC content toward which sequences are evolving according to measured substitution rates) after controlling for recombination rates or ancestral GC content. Data are reported in table 1 and indicate that GC* is significantly higher for both highly recombining and GC-rich sequences compared to their less recombining and GC-poorer counterparts. Yet, different processes seem to explain GC* increase in the two comparisons. All substitution rates increase with recombination, an observation consistent with recombination being mutagenic, as previously suggested [14,53]; in particular, AT->GC substitution rate shows the most marked difference between high vs low recombining regions. Conversely, when recombination was controlled for, we observed a moderate increase of AT->GC rate in GC-rich compared to GC-poor regions, while all other substitution rates (including GC->AT) decrease. This observation, verified in both introns and intergenic spacers (see Additional file 3), rules out the possibility that the confounding effects of extinct recombination hotspots account for substitution rates and increased GC* in GC-rich regions. Indeed, if previously active hotspots had left a molecular signature in GC-rich regions, causing increase in GC content, we would expect substitution rates in GC-rich regions to display a similar trend as those observed in highly recombining regions and, as shown in table 1, this is not the case.

**Table 1:** Substitution rates and GC* in intronic regions

Substitution type	Fixed GC content			Fixed recombination rate		
		
	Low rec.^a^	High rec.^a^	*p*	Low GC	High GC	*p*
A/T -> C/G	0.00068	0.00091	1.9 × 10^-6^	0.00070	0.00075	5.6 × 10^-3^
A/T -> G/C	0.00287	0.00366	1.9 × 10^-6^	0.00275	0.00324	1.9 × 10^-6^
A/T -> T/A	0.00059	0.00065	6.3 × 10^-5^	0.00067	0.00056	5.7 × 10^-6^
C/G -> G/C	0.00096	0.00114	1.9 × 10^-6^	0.00104	0.00101	1.2 × 10^-2^
C/G -> A/T	0.00086	0.00099	1.9 × 10^-5^	0.00108	0.00087	1.9 × 10^-6^
C/G -> T/A	0.00300	0.00336	1.9 × 10^-6^	0.00336	0.00300	1.9 × 10^-6^
CpG -> TpG	0.02427	0.027713	1.9 × 10^-6^	0.03117	0.02352	1.9 × 10^-6^

GC*	0.40750	0.43310	1.9 × 10^-6^	0.37349	0.42967	1.9 × 10^-6^

Number of sites (Mb)	19.42	19.42	-	17.38	17.33	-

^a ^rec.: recombination rate

Still, the data we report here are consistent with selection acting to maintain GC content but also with the presence of mutation biases operating in different GC content regions. In order to evaluate this latter possibility we calculated substitution rates and GC* using either fixed variations or SNPs; while SNPs can reasonably be thought to reflect mutation rates, fixed variations depend on both mutation rates and fixation probabilities. In this case, in order to avoid biases towards high frequency variants, the analysis was restricted to intronic regions deriving from 206 fully resequenced genes (see methods). Also, given the influence, documented above, of recombination on mutation rates, we used only gene regions (1 kb windows) showing low crossover rates.

As shown in table 2, and in agreement with previous findings [54], a very similar (Wilcoxon Rank Sum Tests for paired samples, p = 0.37) intronic GC* is obtained when SNPs are used to infer substitution rates, irrespective of background GC content. Conversely, when fixed variations were taken into account, GC* resulted to be significantly higher for GC-rich than GC-poor sequences. These data suggest that mutation biases, which would be recapitulated by SNP mutations, do not account for the difference in GC* we observe when genomic regions displaying different background GC contents are analyzed; rather, such differences derive from diverse fixation probabilities. These data are therefore fully consistent with the analysis of SNP allele frequency spectra we reported above.

**Table 2:** Substitutions rates and GC* calculated for fixed substitutions and SNPs

Substitution type	Fixed substitutions			SNPs		
		
	Low GC	High GC	*p*	Low GC	High GC	*p*
A/T -> C/G	0.00049	0.00057	4.1 × 10^-1^	0.00020	0.00014	7.6 × 10^-1^
A/T -> G/C	0.00214	0.00257	6.9 × 10^-2^	0.00069	0.00074	7.3 × 10^-1^
A/T -> T/A	0.00049	0.00043	5.0 × 10^-1^	0.00018	0.00014	6.6 × 10^-1^
C/G -> G/C	0.00088	0.00080	5.5 × 10^-1^	0.00031	0.00028	9.0 × 10^-1^
C/G -> A/T	0.00095	0.00075	1.3 × 10^-1^	0.00035	0.00027	2.9 × 10^-1^
C/G -> T/A	0.00275	0.00268	7.3 × 10^-1^	0.00105	0.00109	1
CpG -> TpG	0.03226	0.02081	7.3 × 10^-3^	0.01464	0.01300	6.7 × 10^-1^

GC*	0.35548	0.41816	1.3 × 10^-4^	0.35703	0.38604	3.7 × 10^-1^

Number of sites (Mb)	0.96	0.44	-	0.96	0.44	-

Note: as stated in the text, only regions displaying low recombination rates were analyzed.

Finally, we wished to verify whether analysis of substitution rates and GC* confirmed our above indication that gene expression levels have not been influencing base composition evolution in recent human history. In addition to serving as a useful confirmation, this approach allows analysis of fixed variations at CpGs, which is not feasible using SNP allele frequency spectra (due to recurrent mutations at these dinucleotides); this is relevant to the topic we are addressing since previous authors have indicated that both gene GC content and CpG level correlate with gene expression parameters [19]. Again, we analyzed substitution rates and GC* in genes displaying narrow and wide expression breadth, after controlling for both GC content and recombination rates: we found no significant differences in either substitution rates (including CpG->TpG) or GC* between the two groups of sequences (not shown).

### Local excess of AT->GC fixed variations at recombination hotspots

The possibility that BGC has permanent effects on base composition has recently been questioned [14], being its effect too weak and hotspots too ephemeral. The availability of an outgroup species now allows orientation of substitutions events which accumulated after human/chimpanzee divergence and, therefore, an excess of fixed AT->GC mutation should be observed at recombination hotspots if BGC exerts a strong enough bias. We selected 897 human recombination hotspot on the basis of their size (smaller than 5 kb) and recombination rate (above the 80^th ^percentile of the distribution of all hotspots); in 790 cases both chimpanzee and macaque orthologous regions could be retrieved. As controls, we used 20 samples of randomly selected sequences with a GC content differing less than 1% from that of each hotspot and having its same size. The frequency (Tab. 3) of fixed AT->GC mutations is significantly (Wilcoxon Rank Sum Test for paired samples with Bonferroni correction for multiple tests, maximum *p *= 0.0285) but only slightly (1.08 fold) higher in hotspots compared to control sequences, while no difference is observed for the other substitution types. Yet, no difference in AT->GC fixation was observed when the hotspot 4 kb flanking sequences were compared to their control counterparts. These data are consistent with recombination hotspots having a very small and local effect on GC allele fixation frequency.

**Table 3:** Average frequency of fixed substitutions in recombination hotspots and control regions

Substitution	Hotspot	Control	Maximum *p*	5' hotspot flank	5' control flank	Maximum *p*	3' hotspot flank	3' control flank	Maximum *p*
AT -> GC	0.0051	0.0047	0.0285	0.0048	0.0046	1	0.0048	0.0046	1
GC -> AT	0.0066	0.0066	1	0.0064	0.0065	1	0.0067	0.0064	1
AT -> AT	0.0008	0.0009	1	0.0008	0.0008	1	0.0007	0.0008	1
GC -> GC	0.0013	0.0014	1	0.0013	0.0013	1	0.0014	0.0013	1

*Note: *Frequencies were calculated as number of fixed variation over the number of potentially mutable sites (i.e. for AT>GC the frequency was calculated as the number of substitutions over the total number of AT nucleotides in the human/chimpanzee ancestor). For both hotspots and controls 2 kb flanking sequences were analyzed. *p *values for each comparison (between hotspots and each control sample) were calculated using the Wilcoxon Rank Sum Test for paired samples. The Bonferroni correction for multiple tests was applied and the maximum *p *value is reported.

### Intron GC distribution deviates from neutral expectations

**Figure 3:**
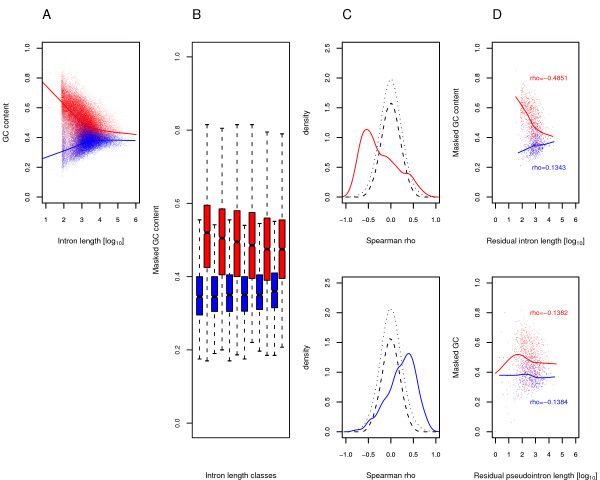
**Analysis of GC content distribution in human introns with different isochoric location**. Isochore definition is as described in [20]. (A) Scatter plot and loess fitting of intron size and GC content in light (blue) and heavy (red) isochores. (B) Analysis of GC_200 _(see text). GC_200 _significantly increases or decreases with residual size (percentile classes are shown) for introns located in heavy (red; breaks in bp = 681, 934, 1309, 1960, 3665) or light (blue; breaks in bp = 810, 1181, 1714, 2638, 5476) isochores, respectively (Kruskall Wallis Test, *p *= 1.3 × 10^-34 ^and 7.9 × 10^-7^, respectively). The number of introns in each size class amounted to 2490 and 1567 for heavy and light isochores, respectively. (C) Distributions of within-gene correlation coefficients. For each gene having more than 15 introns (n = 500 and 1021 for light and heavy isochores, respectively) we calculated correlation coefficients between masked GC content and residual size. Hatched and dotted lines represent envelopes (1^st ^and 99^th ^percentiles, respectively) of correlation coefficient distributions obtained by randomization. (D) Scatter plot and loess fits of GC content over intron size (log_10 _values) for introns (upper panel) and pseudointrons (lower panel). Spearman correlation coefficients (*rho*) are also shown (all *p *values were < 0.01). Introns and pseudointrons were divided on the basis of their isochoric location: blue for light isochores (501 introns-pseudointrons pairs), red for heavy ones (926 pairs).

Finally, we wished to determine whether GC content in human introns conforms to neutral expectations. As shown in figure 3A, human introns located in light and heavy isochores yield two relatively distinct distributions when their GC content is plotted against size; the effect is not due to the presence of transposable elements, since similar trends are observed when GC content is calculated after masking repetitive sequences (see Additional file 4). Yet, a similar relationship is somehow expected: shorter introns are likely to display more extreme GC values due to sampling biases. In order to verify that this is not the sole explanation for our findings, for each intron and after masking for repetitive sequences, we calculated the GC content in a 200 bp window (GC_200_) centered around its median position. In particular, only introns longer than 500 bp were analyzed (in order to avoid splice site constraints) and GC content was calculated only if the 200 central nucleotides were covered by repeats for less than 20% of their sequence. This procedure assures that the same number of intronic nucleotides is used for GC content calculations so that sampling biases (due to extreme variations in intron size) are avoided. Introns from either light or heavy isochores were then grouped in 6 percentile size classes and their GC_200 _analyzed: a significant decrease of GC_200 _with increasing residual intron size (intron size calculated after repeat removal) is observed for introns located in heavy isochores while an increasing trend is evident for those located in GC-poor isochores (Figure 3B).

We speculated that these results might originate from the preferential location of genes with short introns in regions displaying an extreme GC content. Yet, we verified that this is not the case, since introns belonging to the same gene tend to recapitulate the distributions observed above; in particular, introns belonging to genes located in light isochores tend to display an increase in GC content with size; those located in heavy isochores behave in the opposite manner. This is shown in figure 3: we selected genes having more than 15 introns and calculated, for each one, the correlation coefficient between the masked GC content of its intervening regions and their residual size; the distributions of correlation coefficients are shifted to positive and negative values for genes located in light and heavy isochores (Figure 3C), respectively. The significance of this finding was assessed by re-sampling (GC content and intron size were randomly assorted 1000 times for each gene).

All these analyzes have been performed after removal of transposable elements from both GC and size calculations; still, it might be argued that old, unrecognizable transposable elements have contributed to both intronic GC content and size, therefore explaining the observed distributions. In order to verify that this is not the sole explanation for our findings, we analyzed nonrepetitive GC content and residual intron length in intron-pseudointron pairs: old transposable elements gave the same contribution to both intron and pseudointrons (as their insertion predated pseudogene duplication) and therefore, once recognizable transposable elements have been masked, any difference in GC distribution is expected to be accounted for by repeat-independent events. Data are reported in figure 3D and show the homogenization of GC content in short pseudointrons (compared to real ones) located in light or heavy isochores.

It should be noted that many different isochore-identification methods have been described. We therefore verified that the results above were also obtained using IsoFinder [50] isochore definition (see additional file 4 for figures and details); also, the same results are obtained when the gene GC content (rather than isochore attribution) is used to define "light" (average GC content < 0.41) and "heavy" genes (see additional file 4 for figures and details).

In summary, these data indicate that intron GC content and size do not evolve independently; even when possible confounding effects such as size variation, presence of transposable elements and skewed genomic location are taken into account, isochore-specific correlations exist between intron size and GC content. Although there is no theoretic basis to expect it, we verified that no significant difference exists between recombination rates of long and short introns in both heavy and light isochores (not shown, see methods for details). Therefore, the data we report here can hardly be reconciled with a vision whereby BGC alone drives GC content evolution; rather, these finding might be consistent with a role of both base composition and intron size in gene regulation mediated by nucleosome positioning or chromatin conformation, as previously proposed [18,23]. In agreement with this view, it has recently been shown [55] that a considerable amount of human intronic sequence is weakly selected, possibly due to its functioning in chromatin structure and transcription regulation.

## Conclusion

A possible caveat of the data we report here concerns the accuracy of recombination rate measures; the data we used derive from HapMap and refer to crossover rates (and not gene conversion rates); evidences have suggested that, although crossovers and conversions arise from the same recombination-initiating events [54], the ratio of conversions to crossovers can vary among hotspots [55,56]. It is therefore possible that correction for recombination rates leaves a residual; still, there is no a priori reason to expect the residual error to be skewed depending on background GC content. Also, as stated above, analysis of substitution rates in GC-poor vs GC-rich regions do not parallel rates in low-vs high-recombining regions, which would be expected if the same effect (i.e. BGC) were operating in both comparisons. Given this premise and taking into account the analysis of polymorphic repeat insertion and intron GC content distribution, we consider that the more parsimonious explanation for our results is that GC content is subjected to the action of both weak selection and BGC in the human genome with features such as nucleosome positioning or chromatin conformation possibly representing the final target of selective processes. This view might reconcile previous contrasting findings [6,8-13,15-20] and add some theoretical background to recent evidences suggesting that GC content domains display different behaviors with respect to highly regulated biological processes such as developmentally-stage related gene expression [22] and programmed replication timing during neural stem cell differentiation [21].

## Authors' contributions

UP conceived and designed the study, and wrote the paper; GM retrieved data and performed analyzes concerning allele frequency spectra; MF retrieved data and performed analyzes concerning substitution rates; MC retrieved data and performed analyzes concerning retrotransposon insertion polymorphisms; GPC and NB coordinated the study; RC analyzed the relationship between intron size and GC content; MS conceived and designed the study, and wrote the paper.

## Supplementary Material

Additional file 1 analysis of allele frequency spectra for intergenic regions. The figures provided represent quantile-quantile plots of GC->AT and AT->GC derived allele frequencies for 5' and 3' intergenic sequences. Click here for file

Additional file 2 Analysis of fixed versus polymorphic retrotransposon insertions. The figure provides an analysis of polymorphic and fixed retrotransposon relative frequency in different isochores (identified as described in [50]) Click here for file

Additional file 3 substitution rates and GC* in intergenic regions. The data provided represent tables of substitution rates and GC* for 3' and 5' intergenic regions. Click here for file

Additional file 4 Analysis of GC content distribution and size for human introns. the data provide an analysis of intron size and GC content calculated after repeat removal. Also, additional data concerning the relationship between intron size and GC content are shown: in particular, both a different isochore identification procedure was applied and the gene GC content instead of isochore location were used. Click here for file
