# Supplementary material for: Both selective and neutral processes drive GC content evolution in the human genome
Source: BMC Evol Biol. 2008 Mar 27;8:99. doi: 10.1186/1471-2148-8-99 (PMC2292697; doi:10.1186/1471-2148-8-99)
Supplement: Additional file 1 — analysis of allele frequency spectra for intergenic regions. The figures provided represent quantile-quantile plots of GC->AT and AT->GC derived allele frequencies for 5' and 3' intergenic sequences. [file 1471-2148-8-99-S1.pdf]

Additional file 1

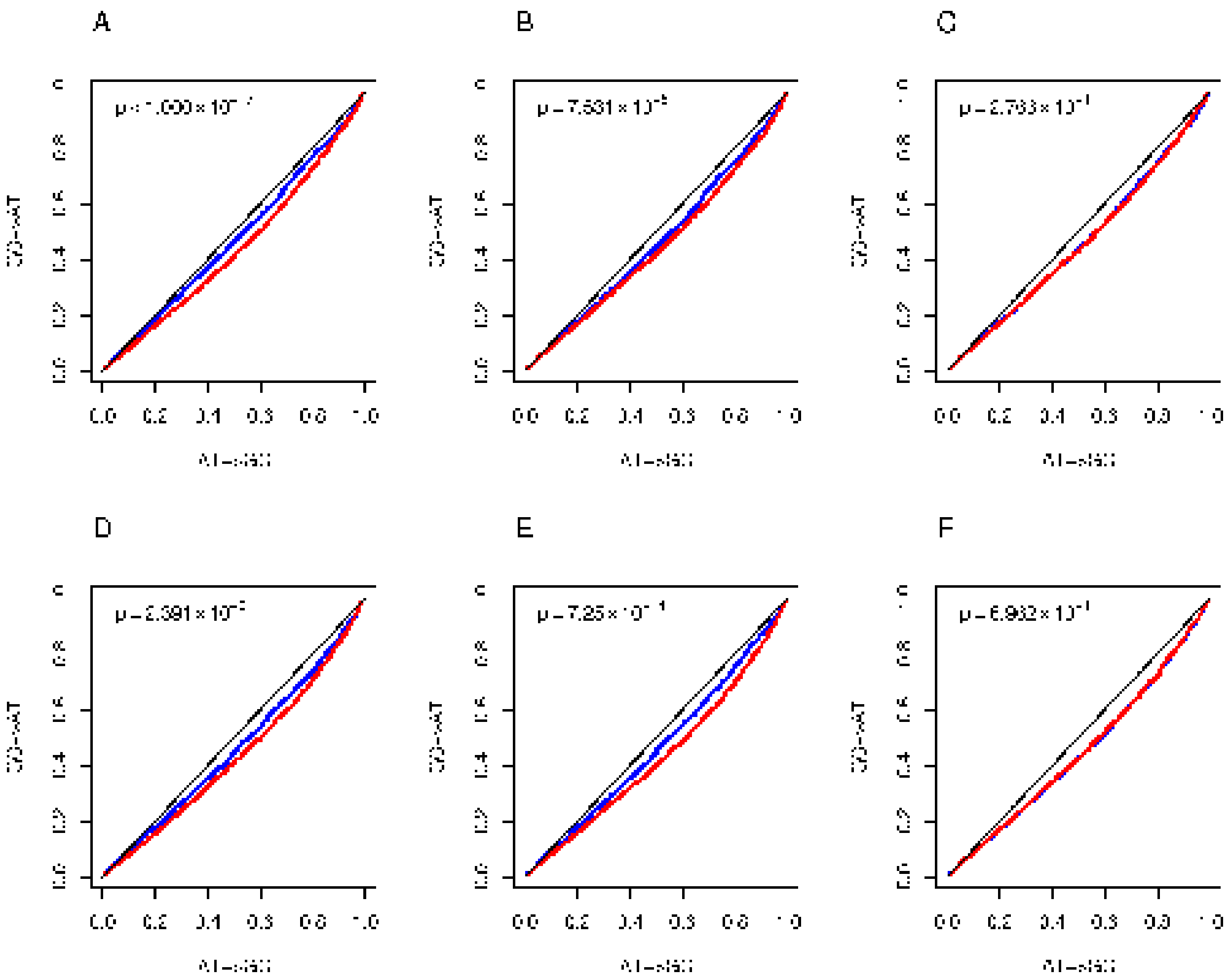

Comparison of allele frequency spectra. Results are for 5' (**A**, **B** and **C**) and 3' (**D**, **E** and **F**) intergenic regions. Gene spacers were defined as 5' or 3' intergenic if the definition was consistent for both flanking genes (i.e. in the case of divergent or convergent flanking gene orientations for 5' ad 3' intergenic, respectively).

(**A, D**) Quantile-quantile plots of GC->AT and AT->GC derived allele frequencies for highly (recombination rate above the 70th percentile) and low recombining intergenic regions (recombination rate below the 30th percentile) after fixing GC content.

(**B, E**) The same as (**A** and **D**), but in this case we fixed recombination rates and compared high vs low GC regions. (**C, F**) The same as (**A** and **D**), but in this case we fixed both GC content and recombination rates in order to compare regions from highly vs lowly expressed genes.

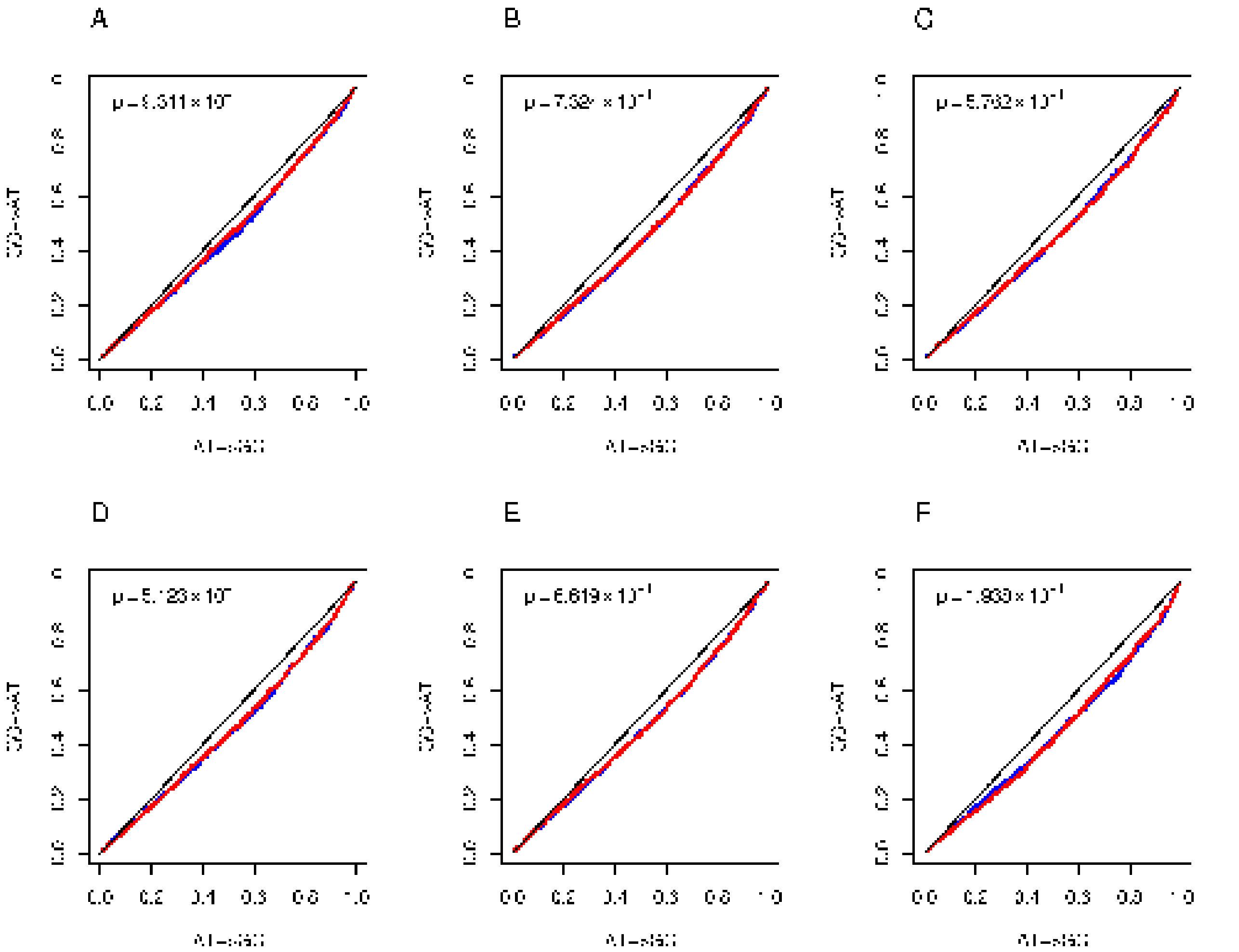

Comparison of allele frequency spectra. Results are for introns (**B**, **E**) and 5' (**A**, **D**) or 3' (**C**, **F**) intergenic regions. Different measure of gene expression were analysed, namely breadth and peak expression.
(**A**-**C**) Quantile-quantile plots of GC->AT and AT-> GC derived allele frequencies for regions deriving from high (red, peak level higher than the 30th percentile) and low (blue, peak level lower than the 30th percentiles) expressed genes after fixing GC content and recombination rate.
(D-F) Quantile-quantile plots of GC->AT and AT-> GC derived allele frequencies for regions deriving from widely (red, breadth ≥ 68 tissues) and narrowly (blue, breadth ≤ 1 tissues) expressed genes after fixing GC content and recombination rate.
