# Supplementary material for: Both selective and neutral processes drive GC content evolution in the human genome
Source: BMC Evol Biol. 2008 Mar 27;8:99. doi: 10.1186/1471-2148-8-99 (PMC2292697; doi:10.1186/1471-2148-8-99)
Supplement: Additional file 2 — Analysis of fixed versus polymorphic retrotransposon insertions. The figure provides an analysis of polymorphic and fixed retrotransposon relative frequency in different isochores (identified as described in [50]) [file 1471-2148-8-99-S2.pdf]

Additional file 2

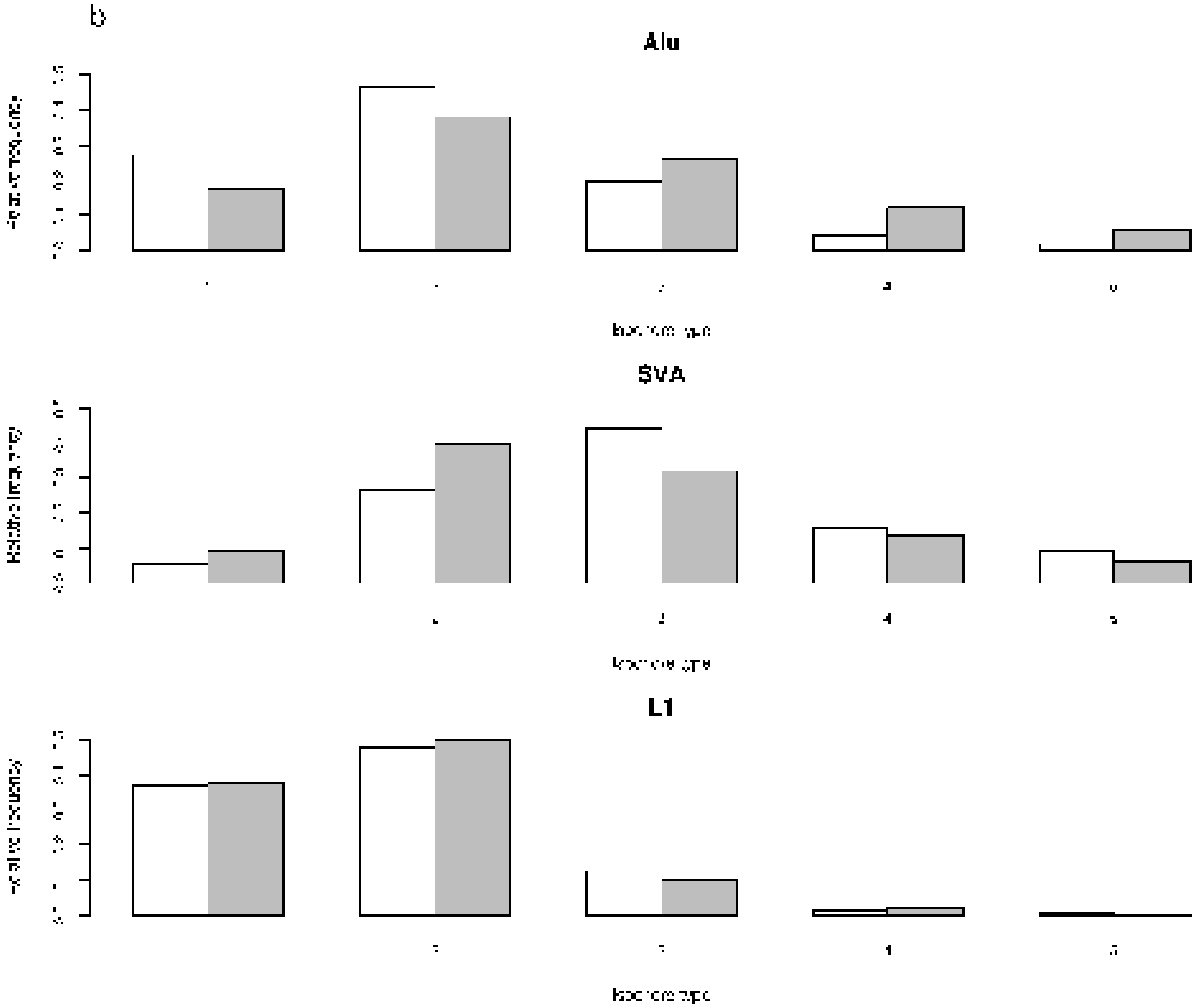

Analysis of polymorphic (white) and fixed (gray) retrotransposon relative frequency in different isochores (isochore definition as in [50], divided in 5 classes according to the following criteria: 0.00 <= C1 < 0.37, 0.37 <= C2 < 0.41, 0.41 <= C3 < 0.46, 0.46 <= C4 < 0.53 and 0.53 <= H3 <= 1.00). Fixed Alus are significantly enriched in heavy isochores compared to polymorphic instances (Chi Square Test, $p<10^{-5}$).
