# Supplementary material for: Both selective and neutral processes drive GC content evolution in the human genome
Source: BMC Evol Biol. 2008 Mar 27;8:99. doi: 10.1186/1471-2148-8-99 (PMC2292697; doi:10.1186/1471-2148-8-99)
Supplement: Additional file 3 — substitution rates and GC* in intergenic regions. The data provided represent tables of substitution rates and GC* for 3' and 5' intergenic regions. [file 1471-2148-8-99-S3.pdf]

Additional file 3

Substitution rates and GC* in 3' intergenic regions.

Substitution type	Fixed GC content			Fixed recombination rate		
	Low rec.[a]	High rec.[a]	*p*	Low GC	High GC	*p*
A/T -> C/G	0.00073	0.00087	5.7×10^{-6}	0.00077	0.00078	5.4×10^{-1}
A/T -> G/C	0.00297	0.00346	1.9×10^{-6}	0.00292	0.00330	1.9×10^{-6}
A/T -> T/A	0.00063	0.00069	2.1×10^{-2}	0.00070	0.00056	2.6×10^{-4}
C/G -> G/C	0.00104	0.00110	6.3×10^{-2}	0.00103	0.00102	8.9×10^{-1}
C/G -> A/T	0.00108	0.00111	3.2×10^{-1}	0.00123	0.00095	1.9×10^{-6}
C/G -> T/A	0.00327	0.00357	3.8×10^{-6}	0.00366	0.00316	3.8×10^{-6}
CpG -> TpG	0.02790	0.02976	3.5×10^{-3}	0.03456	0.02348	1.9×10^{-6}
GC*	0.39192	0.40835	3.9×10^{-4}	0.36860	0.42301	1.9×10^{-6}
Number of sites (Mb)	8.38	8.37	-	6.26	6.24	-

[a] rec.: recombination rate; the number of 1kbseq couples were 8453 and 6303 for fixed GC content and fixed recombination rate, respectively

Substitution rates and GC* in 5' intergenic regions.

Substitution type	Fixed GC content			Fixed recombination rate		
	Low rec.[a]	High rec.[a]	*p*	Low GC	High GC	*p*
A/T -> C/G	0.00072	0.00086	1.9×10^{-6}	0.00076	0.00079	1.0×10^{-1}
A/T -> G/C	0.00295	0.00353	1.6×10^{-6}	0.00303	0.00336	1.9×10^{-6}
A/T -> T/A	0.00064	0.00071	1.3×10^{-5}	0.00076	0.00061	3.8×10^{-6}
C/G -> G/C	0.00102	0.00113	2.6×10^{-5}	0.00108	0.00106	4.1×10^{-1}
C/G -> A/T	0.00110	0.00113	7.5×10^{-2}	0.00131	0.00101	1.9×10^{-6}
C/G -> T/A	0.00335	0.00355	1.3×10^{-4}	0.00375	0.00329	1.9×10^{-6}
CpG -> TpG	0.02667	0.03045	1.9×10^{-6}	0.03611	0.02341	1.9×10^{-6}
GC*	0.38669	0.41076	1.9×10^{-6}	0.36732	0.41879	1.9×10^{-6}
Number of sites (Mb)	28.00	28.02	-	18.88	18.85	-

[a] rec.: recombination rate; the number of 1kbseq couples were 28248 and 19025 for fixed GC content and fixed recombination rate, respectively
