# Supplementary material for: Both selective and neutral processes drive GC content evolution in the human genome
Source: BMC Evol Biol. 2008 Mar 27;8:99. doi: 10.1186/1471-2148-8-99 (PMC2292697; doi:10.1186/1471-2148-8-99)
Supplement: Additional file 4 — Analysis of GC content distribution and size for human introns. the data provide an analysis of intron size and GC content calculated after repeat removal. Also, additional data concerning the relationship between intron size and GC content are shown: in particular, both a different isochore identification procedure was applied and the gene GC content instead of isochore location were used. [file 1471-2148-8-99-S4.pdf]

Additional file 4

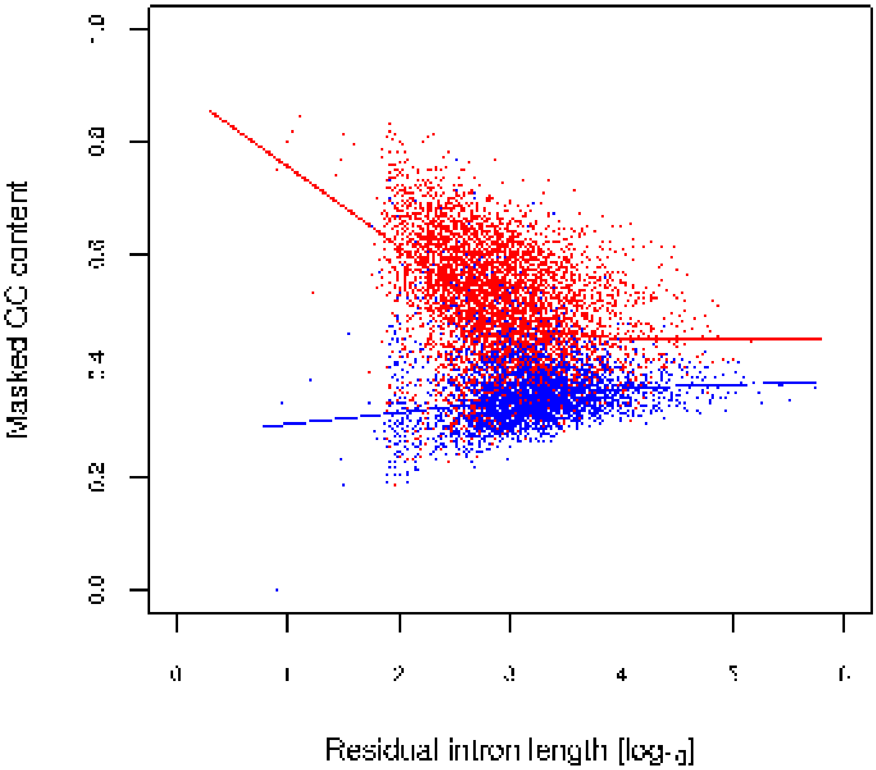

Scatter plot and loess fitting of residual intron size (after repeat removal) and masked GC content in light (blue) and heavy (red) isochores (as defined in Ref [20] in the manuscript).

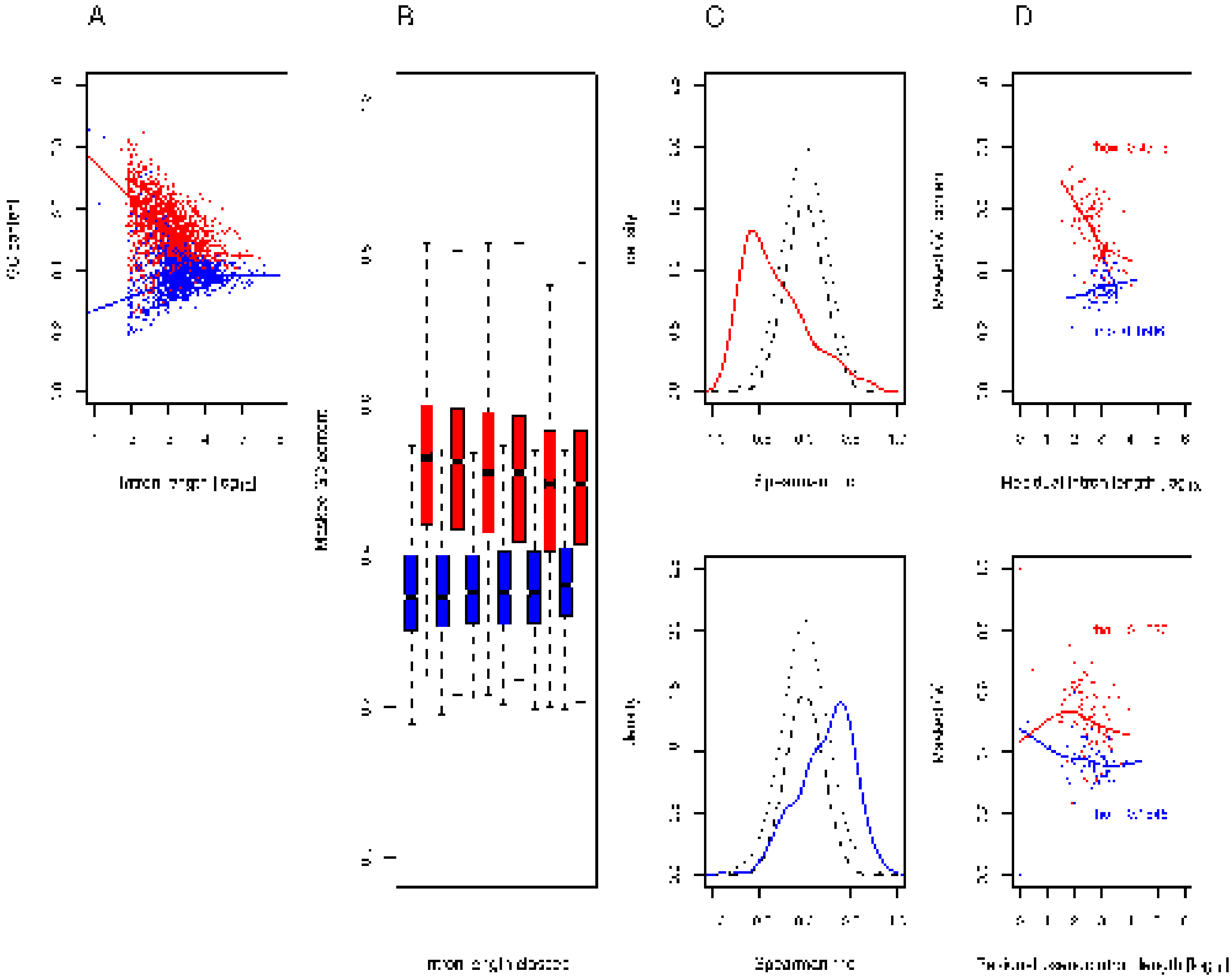

Analysis of GC content distribution in human introns with different isochoric location (isochore definition as in Ref [50], considered heavy if GC>=0.41, light if not). (A) Scatter plot and loess fitting of intron size and GC content in light (blue) and heavy (red) isochores. (B) GC content was calculated in 200 bp windows (GC200) centered around the median position of each intron after masking repetitive sequences. GC200 significantly increases or decreases with residual size (percentile classes are shown) for introns located in heavy (red; breaks in bp = 669, 909, 1267, 1856, 3454) or light (blue; breaks in bp = 805, 1172, 1693, 2626, 5418) isochores, respectively (Kruskall Wallis Test, p $=4.7\times10^{-23}$ and 3.6×10^{-5}, respectively). Only introns longer than 500 bp were analyzed (in order to avoid splice site constraints) and GC content was calculated only if the 200 central nucleotides were covered by repeats for less than 20% of their sequence. The number of introns in each size class amounted to 1615 and 1143 for heavy and light isochores, respectively. (C) Distributions of within-gene correlation coefficients. For each gene having more than 15 introns (n = 319 and 650 for light and heavy isochores, respectively) we calculated correlation coefficients between masked GC content and residual size. Hatched and dotted lines represent envelopes (1st and 99th percentiles, respectively) of correlation coefficient distributions obtained by randomization (for each gene's introns masked GC content and residual size were randomly assorted 1000 times). (D) Scatter plot and loess fits of GC content over intron size ($\log_{10}$ values) for introns (upper panel) and pseudointrons (lower panel). Only gene-pseudogene couples located in the same isochore type were analyzed (see methods). Spearman correlation coefficients (rho) are also shown (all p values were < 0.01). Introns and pseudointrons were divided on the basis of their isochoric location: blue for light isochores (415 introns-pseudointrons pairs), red for heavy ones (624 pairs).

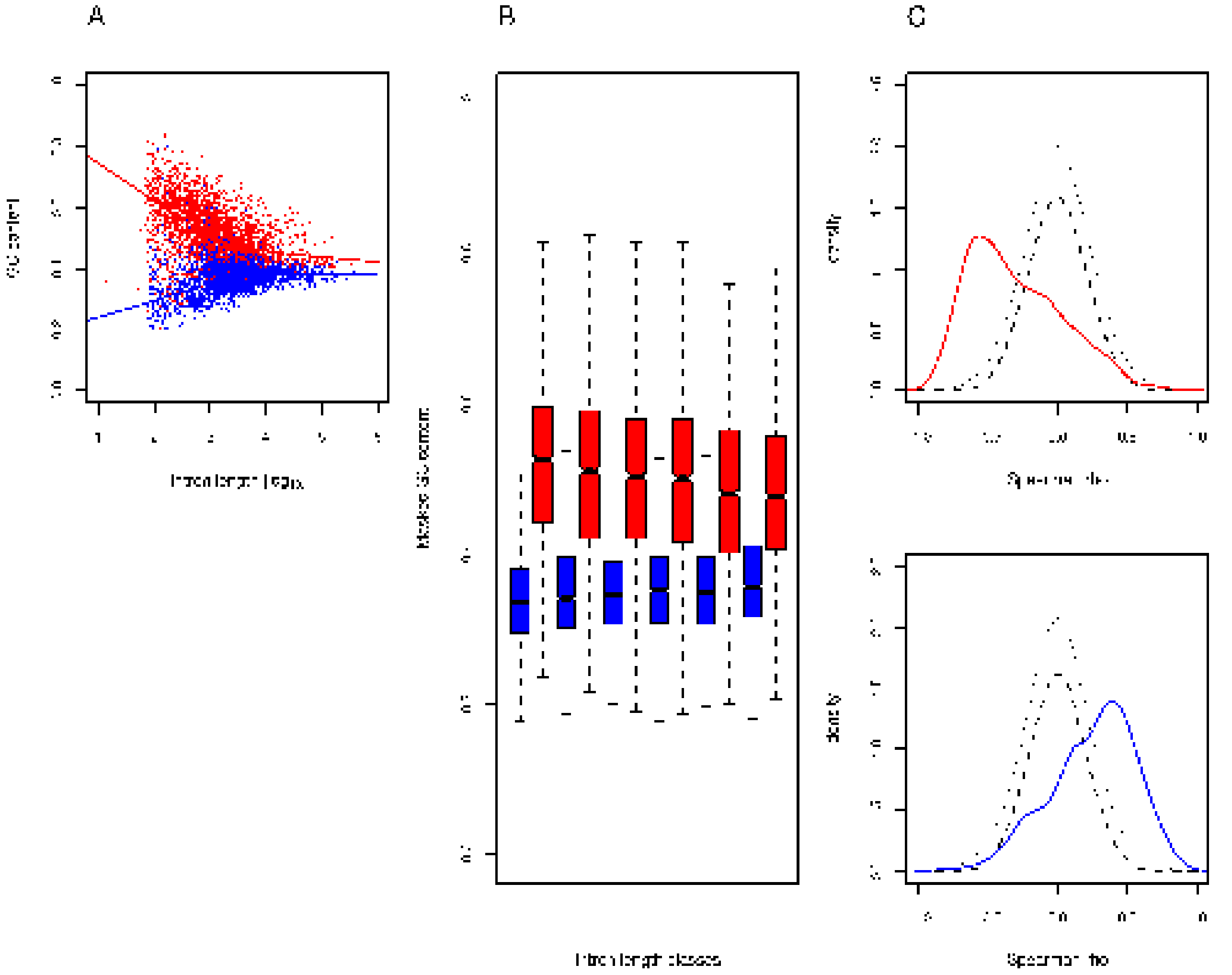

Analysis of GC content distribution in human introns. Genes were divided in “light” and “heavy” on the basis of their average GC content (GC content of 0.41 was used as the cutoff). (A) Scatter plot and loess fitting of intron size and GC content in light (blue) and heavy (red) genes. (B) GC content was calculated in 200 bp windows (GC200) centered around the median position of each intron after masking repetitive sequences. GC200 significantly increases or decreases with residual size (percentile classes are shown) for introns from high GC (red; breaks in bp = 682, 943, 1338, 2032, 3961) or low GC (blue; breaks in bp =818, 1202, 1767, 2769, 5994) genes respectively (Kruskall Wallis Test, $p < 5.4x10^{-48}$ and $1.4x10^{-21}$, respectively). Only introns longer than 500 bp were analyzed (in order to avoid splice site constraints) and GC content was calculated only if the 200 central nucleotides were covered by repeats for less than 20% of their sequence. The number of introns in each size class amounted to 2633 and 2043 for heavy and light genes. (C) Distributions of within-gene correlation coefficients. For each gene having more than 15 introns (n = 728 and 1036 for low and high gene GC, respectively) we calculated correlation coefficients between masked GC content and residual size. Hatched and dotted lines represent envelopes (1st and 99th percentiles, respectively) of correlation coefficient distributions obtained by randomization (for each gene's introns masked GC content and residual size were randomly assorted 1000 times).
